# Socioeconomic inequality in financial hardship in accessing quality healthcare services in Ethiopia: a community-based cross-sectional study

**DOI:** 10.3389/fpubh.2025.1484671

**Published:** 2025-03-26

**Authors:** Yawkal Tsega, Zelalem Birhan, Kidist Adamu

**Affiliations:** ^1^Department of Health System and Management, School of Public Health, College of Medicine and Health Sciences, Wollo University, Dessie, Ethiopia; ^2^Department of Psychiatry, School of Medicine, College of Medicine and Health Sciences, Wollo University, Dessie, Ethiopia

**Keywords:** socioeconomic inequality, catastrophic health expenditure, impoverishing health expenditure, concentration index, Ethiopia

## Abstract

**Background:**

The 2030 Agenda for Sustainable Development aims to ensure that no one is left behind in health. However, the high magnitude of catastrophic health expenditure (CHE) and impoverishing health expenditure (IHE) remain global challenges. The financial hardship caused by healthcare has not been extensively studied in Ethiopia to date. Therefore, this study aimed to assess socioeconomic inequality in financial hardship and its determinants among households in the South Wollo zone, Ethiopia.

**Methods:**

This cross-sectional study surveyed 845 households in the South Wollo zone from 1 May to 31 May 2023. Financial hardship was measured using the IHE and CHE metrics. The households were considered to experience IHE if their health expenditure pushed them below a poverty line of $2.15 (ETB 118.25) and considered to experience CHE if their health expenditure exceeded 10% of their total expenditure. Costs were estimated using prevalence-based and patient-perspective approaches. STATA version 17.0 was used for data management and analysis. We used the cixr and lorenz estimate STATA commands to estimate the concentration index (CIX) and generate the concentration curve (CC), respectively. An adjusted odds ratio (AORs) with a 95% confidence interval and a *p*-value of <0.05 were used to determine statistical significance.

**Results:**

The CIX for wealth status was −0.17 (CI: −0.23, −0.11), with a *p*-value <0.001, indicating significant socioeconomic inequality in financial hardship of healthcare. The incidence of CHE was ~30% (95%CI; 26.91–33.16%) at the 10% threshold, while the incidence of IHE was ~4% at the $2.15 poverty line. Significant determinants of CHE included the poorest wealth status (AOR: 4.80, CI: 2.61–8.86), older age of the household head (AOR: 3.40, CI: 1.52–7.60), lack of insurance (AOR: 2.70, CI: 1.67–4.38), chronic illnesses (AOR: 5.12, CI: 3.24–8.10), being widowed (AOR: 4.30, CI: 1.27–14.57) or divorced (AOR: 6.45, CI: 1.89–21.10) in terms of marital status of the household head, and seeking traditional healthcare (AOR: 2.47, CI: 1.60–3.81).

**Conclusion:**

This study revealed that there was significant inequality in financial hardship of health expenditure across household wealth categories. The incidences of CHE and IHE were higher. The wealth status of the household, insurance status, marital status of the household head, chronic illness, and seeking traditional healthcare were the key determinant factors of CHE. Therefore, policymakers should focus on underprivileged households to ensure effective healthcare financial risk protection (FRP).

## Background

The 2030 Sustainable Development Agenda is committed to the promise of leaving no one behind in health (1–3). The best way to achieve this goal is through the implementation of universal health coverage (UHC), which guarantees that every person has access to quality essential health services, regardless of their location or time of need, and without the risk of experiencing financial hardship (3–6). Financial risk protection (FRP) is essential for achieving UHC. However, in many low-income countries, health systems often fail to protect against high out-of-pocket (OOP) health spending, leading to significant financial hardship related to healthcare (7).

Since the 2005 World Health Assembly, many countries have pledged to safeguard households from the financial risks associated with catastrophic out-of-pocket (OOP) health expenditures (7–9). In addition, many member states of the World Health Organization (WHO) have restructured their healthcare systems with the goal of achieving UHC by 2030. This transformation includes implementing measures for FRP for households and ensuring the provision of equitable essential health services within their respective countries (7, 10).

Catastrophic health expenditure (CHE) and impoverishing health expenditure (IHE) are the two metrics used to measure the financial hardship of healthcare (3, 6, 11). The Sustainable Development Goal indicator 3.8.2 (SDG indicator 3.8.2) is tracked by the United Nations member states using IHE and CHE (3, 11, 12). CHE and IHE are evaluated when a household’s health costs exceed a specific threshold (ranging from 10 to 40%) of its income or total expenditure and when these expenses push the household below a certain poverty line, respectively (3, 11, 13).

The tracking UHC Global Monitoring Report stated that approximately 344 million people live in extreme poverty (3) and approximately 2 billion people faced financial hardship in 2019 as a result of paying for healthcare services (3). Moreover, nearly half of the world’s population, 4.5 billion people, did not have access to basic healthcare service coverage (3).

The number of individuals experiencing CHE has risen since 2000, surpassing 1 billion globally by 2019 (6, 11). This prevents households from affording basic needs such as food and education. The incidence of CHE (using the 10% threshold level) increased from 9.6% in 2000 to 12.6% in 2015 and further to 13.5% in 2019. Similarly, the number of people experiencing CHE increased by 76% from 588 million in 2000 to 1.04 billion in 2019 (4, 6, 9, 11, 14). However, the magnitude of IHE decreased by 80% at the extreme poverty line between 2000 and 2019 (6). The global incidence of IHE, using the $2.15 per day extreme poverty line, decreased from 22.2% in 2000 to 4.4% in 2019 (4, 8, 11, 14).

Although the Ethiopian health system has committed to strengthening FRP for households through community-based health insurance, fee waiver systems, and exempted services, healthcare financing remains largely dependent on OOP (i.e., 30% of total health expenditure) payments (11, 13, 15). This leads to inequitable healthcare utilization, with poor households being the most affected group. Households are experiencing financial hardships of healthcare due to unpredictable and poorly harmonized healthcare expenditure systems. Factors such as wealth status, residence, working conditions of adults, the presence of vulnerable and older members in the household, family size, age, employment status, educational status, and the sex of the household head all contribute to the likelihood of households experiencing CHE (11, 13, 16, 17).

Furthermore, the high dependence on OOP healthcare expenditure usually leads to CHE, disproportionately affecting those in lower socioeconomic categories and worsening socioeconomic inequality in financial hardship of health expenditure (3, 11). This study aimed to uncover the extent of socioeconomic inequality, the magnitude of CHE and IHE, and their determinant factors and to inform policymakers in designing policies and strategies aimed at enhancing FRP and achieving UHC.

To the best of our knowledge and based on a review of the literature, little has been studied regarding socioeconomic inequality in financial hardship in accessing quality healthcare in Ethiopia in general and in the South Wollo zone in particular. While several studies have been conducted on CHE and its associated factors, they are often limited to specific institutions or disease types (18–20). This comprehensive study evaluated socioeconomic inequality and all health expenditures for various health services, providing crucial evidence for policymakers to design strategies that ensure FRP for households in Ethiopia and other similar contexts. Therefore, this study aimed to assess socioeconomic inequality, the incidence, and the determinants of financial hardship in accessing quality healthcare services in the South Wollo zone.

## Methods

### Study design, setting, and period

**Figure 1:**
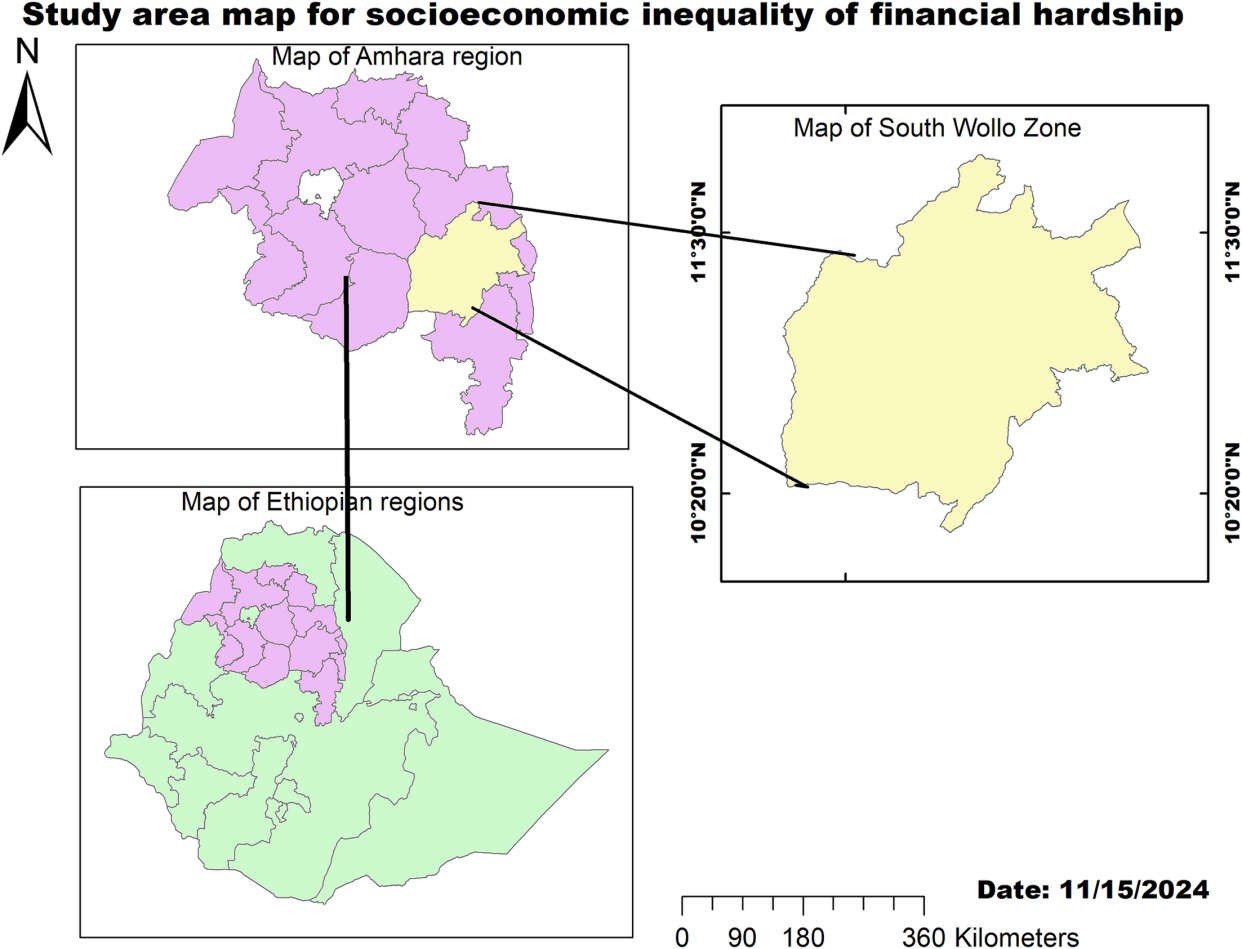
Map of the study area showing socioeconomic inequality in financial hardship in the South Wollo zone, Northeast Ethiopia, 2023.

This cross-sectional study was conducted in the South Wollo zone from 1 May to 31 May 2023. The South Wollo zone is located in the Amhara region of Ethiopia, with its capital in Dessie. It is 401 km away from the capital of Ethiopia, Addis Ababa (Figure 1). According to the 2023 report from the Ethiopian Statistical Service, South Wollo has a total population of 3,387,395 and households of 804,607, resulting in an average of 4.21 persons per household.^1^ The 2007 Census indicated that this zone has an area of 17,067.45 square kilometers, with a population density of 147.58. Moreover, the South Wollo zone has 10 primary hospitals, 129 health centers, 523 health posts, 134 private primary clinics, 47 medium clinics, and 1 non-governmental health facility that provides preventive and curative services to people.

### Population and eligibility criteria

All households in the south Wollo zone were considered the source population, and all households in the randomly selected districts and city administrations, such as Kutaber district, Dessie Zuria district, Legambo district, Dessie city administration, Kombolcha city administration, and Hayk city administration, were considered the study population. All households residing for more than 6 months in the randomly selected districts and city administrations were included in the study, while household heads who were ill, unable to hear, or with mental incompetency were excluded from the study.

### Variables

#### Outcome variable

Catastrophic Health Expenditure was the main outcome variable for this study. Moreover, Impoverishing Health expenditure, one of the metrics of financial hardship, was also considered an outcome variable.

#### Explanatory variables

The explanatory variables were grouped into three categories: socioeconomic and demographic factors (sex of the household head, age of the household head, religion of the household head, ethnicity, marital status of the household head, family size, wealth status, the presence of under-five children in the household, educational status, and occupational status), health-related variables (the presence of chronic health conditions, referral history, traditional healthcare seeking, and type of health institution), and coping strategies (insurance status, selling assets, saving, borrowing money, and social support).

### Operational definitions

#### Catastrophic health expenditure

In this study, CHE was defined as households spending more than 10% of their total reported expenditure on healthcare services (3, 4, 13).

#### Healthcare expenditure

Healthcare expenditure was defined as the total household expenditure related to healthcare, which included direct medical costs, direct non-medical costs, and indirect healthcare costs (3, 13).

#### Impoverishing health expenditure

In this study, IHE was considered when households were pushed below the $2.15 (ETB 118.25) per day extreme poverty line, as defined by the World Bank, due to their healthcare expenditure (3).

#### Poverty gap (poverty gap index)

This measures how far households are from the poverty line and the intensity of poverty (11, 13).

#### Wealth index

The wealth index is a composite measure of a household’s cumulative living standard. The wealth index is calculated using easy-to-collect data on a household’s ownership of selected assets (such as televisions and bicycles), materials used for housing construction, and types of water access and sanitation facilities (11, 21, 22).

### Outcome and related variables measurement

#### Types of costs and their costing methods

Both the patient and the caregiver’s direct medical and non-medical expenses were estimated (23–25). To determine the direct costs of healthcare services, a bottom-up (micro) costing approach, based on the average cost of healthcare services, was used (11, 26, 27). This approach involves a detailed enumeration and costing of every input used in the treatment of a patient (28). The annual average direct medical and non-medical expenditures for each household were estimated by summing all self-reported healthcare expenditures from May 2022 to May 2023.

Indirect costs, estimated using a human capital approach, in this study included the costs of lost days (absenteeism from their job) both for the patient and caregiver. For workers (payroll employees and merchants), the monetary value of lost days was calculated by multiplying the number of lost days by the reported personal daily income (monthly income divided by 30). For non-payroll households, such as those with farmers, their reported annual household income was used to estimate the cost of lost days.

#### Measurement of catastrophic and impoverishing health expenditures

To measure CHE and IHE, the Wagstaff and van Doorslaer approach, which was published in 2003, was employed (29). When a household’s health expenses exceed a certain threshold level of their total household expenditures, income, or non-food expenses, CHE is considered. The threshold used to calculate CHE is arbitrary and ranges from 10 to 40% (11, 13, 29).

To estimate the catastrophic headcount, which is the percentage of households incurring catastrophic expenditures, we defined **T**_**HE**_ as the total annual health expenditures for household i, **T**_**E**_ as the total annual expenditure for household i, and **F**_**E**_ as the food expenditures for household i (11, 13).

A household was considered to have CHE if **T**_**HE**_**/T**_**E**_ surpassed a specified threshold, Z (in this study, a 10% threshold was used) (11, 13).

The catastrophic headcount (Hc) is defined as Equation 1:


(1) $$H_{c}=\frac{1}{N}\overset{N}{\underset{i=1}{\sum }}E_{i}$$

Where N is the sample size and E_i_ equals 1 if **T**_**HE**_**/T**_**E**_ > z and 0 otherwise.

The headcount does not reflect the amount by which households exceed the threshold. Therefore, we used the catastrophic expenditure overshoot, which captures the average degree to which health expenditures (as a proportion of total expenditure or non-food expenditure) exceed the threshold Z (11). The overall overshoot (O) is defined as Equation 2:


(2) $$O=\frac{1}{N}\overset{N}{\underset{i=1}{\sum }}O_{i}$$

Where O_i_ = E_i_ ((T_HE_/T_E_) − z).

Where E_i_ = ((T_HE_/T_E_)-z) if (T_HE_/T_E_) > z, and 0 otherwise.

The incidence (headcount) and intensity (overshoot) of catastrophic expenditures are related through the mean positive overshoot (MPO), which captures the intensity of catastrophic expenditures. It is defined as the overshoot divided by the headcount (Equation 3):


(3) $$MPO=\frac{O}{H};O=H^{*}MPO$$

Wagstaff and van Doorslaer also described methods to adjust poverty measures based on household expenditure net of OOP spending on healthcare (11, 29). The three measures of poverty include the following:

1 **Poverty headcount**: The proportion of households living below the poverty line (the $2.15 per day extreme poverty line used in this study) (Equation 4);

(4) Hpovpre=1N∑i=1NPipre=μPpre

Where $H_{pov}^{pre}$ is the poverty headcount before health payments, and P_i_^pre^ = 1 if X_i_ < PL and zero otherwise.

2 **Poverty gap**: This refers to the aggregate of all shortfalls from the poverty line (Equation 5).

(5) Gpovpre=1N∑i=1Ngipre=gμpre

Where $G_{pov}^{pre}$ is the prepayment poverty gap, and g_i_^pre^ = PL-X_i_ if PL > X_i_ and zero otherwise.

3 **Normalized poverty gap (*N*** $G_{pov}^{pre}$ **) or poverty gap index**: This is obtained by dividing the poverty gap by the poverty line (Equation 6).

(6) $$NG_{pov}^{pre}=\frac{G_{pov}^{pre}}{\mathrm{PL}}$$

This study used a poverty line of $2.15 per person per day, which was converted to ETB based on the average exchange rate (1 USD = ETB 55) from September 2022 to August 2023, to estimate poverty levels before and after healthcare expenditure.

The measures of poverty impact (PI^H^) from health expenditure are simply defined as the difference between the prepayment and post-payment measures (Equation 7), i.e.,


(7) $$PI^{H}=H_{pov}^{\mathrm{post}}-H_{pov}^{pre}$$

#### Inequality measurement

Across socioeconomic categories of the households, the concentration curve (CC) and concentration index (CIX) in their relative form (without correction) were employed to assess socioeconomic inequality in terms of CHE and IHE (30, 31). The CIX in this study represented horizontal inequity as each household in the study was assumed to have an equal burden of catastrophic and impoverishing health expenditures.

During the construction of the CC, the cumulative proportion of the households ranked by wealth status (poorest first) was plotted on the *x*-axis against the cumulative incidence of impoverishing and catastrophic health expenditures on the *y*-axis, each separately. A 45-degree slope from the origin represents perfect equality. If the CC overlaps with the line of equality, the incidence of CHE and IHE is equal across the households.

However, if the CC lies above or below the line of equality, then inequality in the incidence of CHE and IHE exists, with the curve slanting toward the households in either the low or high socioeconomic category. The further the CC is from the line of equality, the greater the degree of inequality. To evaluate the extent of wealth-related inequality, the CIX was estimated. The CIX is twice the area between the line of equality and CC. The CIX value ranges from −1 to +1. A positive CIX value implies that the incidence of CHE/IHE is concentrated among higher socio-economic groups (pro-rich). When the incidence of CHE/IHE is evenly distributed across socioeconomic classes, the CIX equals zero. In contrast, a negative value of the CIX indicates that the incidence of CHE/IHE is primarily concentrated in lower socioeconomic groups (pro-poor). The estimation of the CIX was made using the “convenient covariance” formula described by O’Donnell et al. (30), as shown in Equation 8,


(8) $$CIX=\frac{2}{\mu }cov\left(h,r\right)$$

Where h is the health variable, μ is its mean, and r = i/N represents the fractional rank of individual i in the living standards distribution, with i = 1 for the poorest and i = N for the richest. The user-written STATA commands lorenz estimate (32) and cixr (33) were used to generate the CC and estimate the CIX, respectively.

### Sample size determination

The single population proportion formula was used to estimate the sample size, assuming a 50% proportion of CHE at a 10% threshold level, a confidence level of 95%, a degree of precision of 5%, and a non-response rate of 10%. The total sample size was calculated as follows:


$$n=\frac{\left(Z_{@/2}\right)2*p\left(1-q\right)\;}{d^{2}}=\frac{\left(1.96\right)2*o.5\left(1-0.5\right)}{0.05^{2}}=384$$

Where *p* = 50%.

d = 0.05 (degree of precision), and Zα/2 at the 95% confidence level = 1.96.

By adding a 10% non-response rate and a design effect of 2, the final sample size was calculated as 768 + 0.1*768 = 845 households.

### Sampling method and procedures

**Figure 2:**
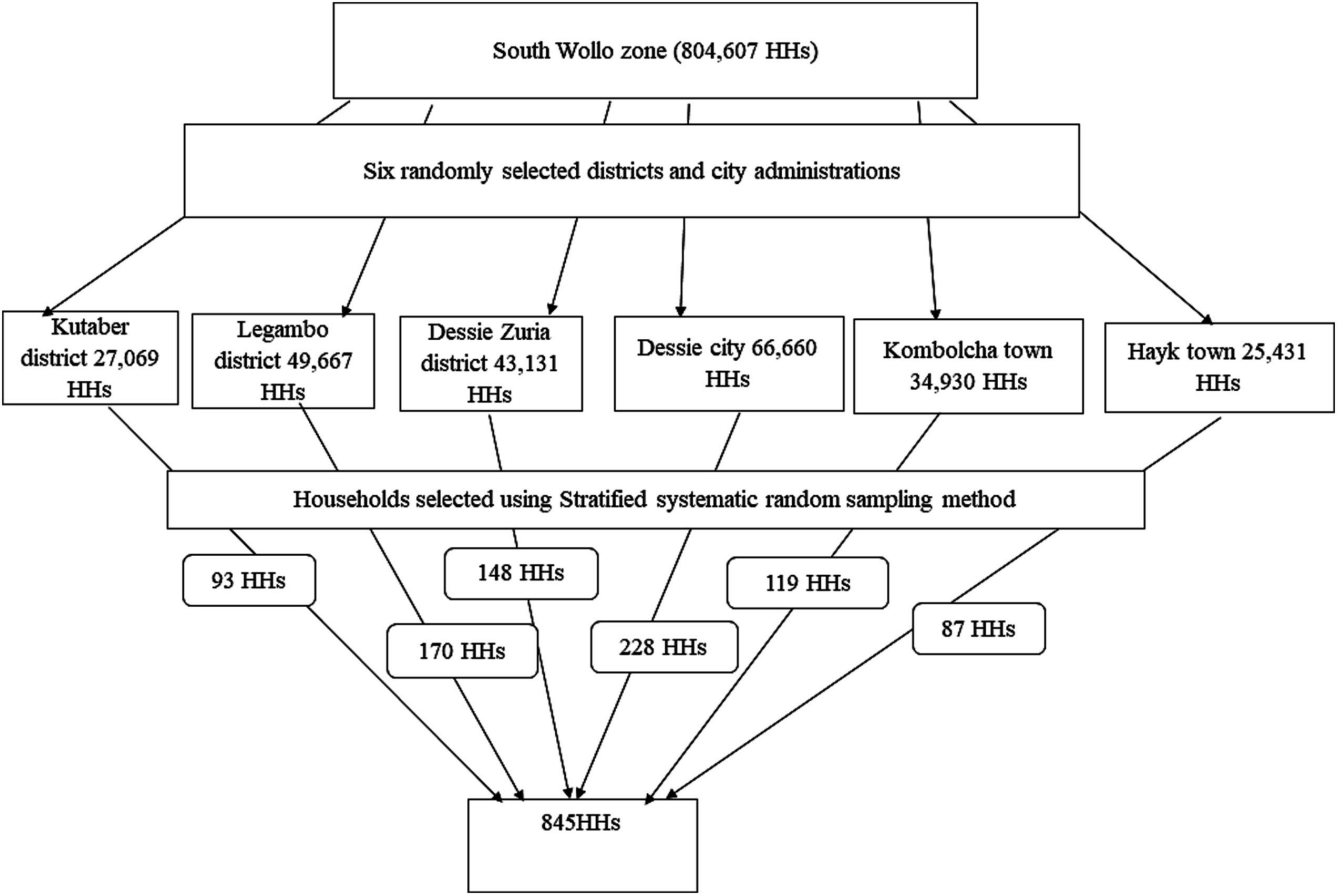
Diagrammatic depiction of the sampling procedure, South Wollo zone, Northeast Ethiopia, 2023.

Three rural districts (Dessie Zuria district, Legambo district, and Kutaber district) and three city administrations (Dessie, Kombolcha, and Hayk city administrations) were selected randomly using the lottery method. There are 246,888 households in the randomly selected districts and city administrations. The sampled households were proportionally allocated to each randomly selected district and city administration. A total of 845 households were selected using the systematic random sampling method in each stratum and district. The sampling procedure is depicted in Figure 2.

### Survey instruments and data collection procedures

A structured questionnaire was developed following a thorough review of relevant literature. The survey instrument comprised various categories aimed at gathering data on the socioeconomic and demographic characteristics, the health profile of the households, their associated characteristics, total household expenditures, total healthcare expenditures, and coping strategies in the face of financial hardships. The data collection team comprised six experienced individuals with a bachelor’s degree in public health and three supervisors with a master’s degree in public health.

Data regarding total annual expenditures, covering the period from May 2022 to May 2023 and including both healthcare and other household expenses, were collected from the head of each selected household. Summations were made for each expenditure category—healthcare, food, and non-food expenditures—culminating in the evaluation of total annual health expenditure, total annual food expenditure, total annual non-food expenditure, and total annual household expenditure. These values were then used as denominators in calculating the incidence of CHE and IHE.

In addition, the questionnaire included wealth index assessment variables adapted from the Ethiopian Mini Demographic and Health Survey 2019, tailored for both urban and rural settings (34). The household head was asked 35 questions evaluating aspects such as sanitation facilities, sources of drinking water, housing conditions, and ownership of durable assets. The wealth index was constructed by considering the durable assets owned by households, household sanitation facilities, sources of drinking water, and housing conditions. Each asset was assigned a weight or factor score based on its perceived indication of wealth and standardized to a normal distribution. A standardized score was then assigned to each household based on their possession of these assets, and the scores were summed to determine the total household score. The households were ranked based on these scores and divided into five groups, each representing 20% of the population, known as quintiles. The lowest quintile represented the poorest households, while the highest quintile represented the richest households. The use of wealth quintiles is preferred over income or consumption in assessing long-term economic status, and it is also more practical to implement (11, 13, 34).

### Data management and analysis

The collected data were checked for completeness, entered into EpiData version 4.6, and exported to STATA version 17.0 for analysis. Descriptive statistical analyses, such as summary statistics, frequencies, and percentages, were conducted, along with bivariable and multivariable logistic regression analyses. In the bivariable logistic regression analysis, variables with a *p*-value of <0.2 and a confidence interval of 95% were eligible for inclusion in the multivariable logistic regression analysis. The overall goodness-of-fit of the binary logistic regression model was assessed using the Hosmer and Lemeshow test (*p* = 0.605). The assumptions of binary logistic regression, such as multicollinearity and the presence of outliers, were assessed for this model. Adjusted odds ratios (AOR) with 95% confidence intervals were computed to assess the strength of the association. Statistical significance was considered at a *p*-value of <0.05 for all analyses.

We constructed the wealth index using principal component analysis (PCA) with STATA version 17.0. We included 35 variables assessing various aspects of household living standards. Variables that appeared in over 95% of the households were excluded, as they offered little differentiation in wealth status. For example, if nearly all households had access to a particular sanitation facility, it would not help us distinguish wealth levels effectively. Similarly, variables that were present in fewer than 5% of the households were also excluded, as they lacked sufficient variability to provide useful insights. Similarly, in the PCA output’s correlation matrix, values below 0.1 and above 0.9 were excluded from the wealth index construction. Finally, 15 variables were used to construct the wealth index. The first principal component of the composite variables, which explains the largest proportion of the total variance, was used to construct and rank the wealth index of the households in ascending order across the five quintiles.

### Data quality assurance

The structured questionnaire was first prepared in English and then translated into Amharic by language experts. Then, 3 days of training were provided to the data collectors and supervisors on the overall structure of the questionnaire, how to collect data, and how to approach the respondents. Before the actual data collection, pretesting was conducted on 5% (43 households) of the sample size in Kalu district. The data collectors were closely supervised, and the data were checked for completeness on a daily basis.

## Results

### Socioeconomic and demographic factors

A total of 825 household heads participated in this study, resulting in a response rate of 97.63%. Among these households, 574 (69.58%) were headed by male individuals. The mean age of the household heads was 43.36 years, with a standard deviation of 14.28 years. A total of 343 (41.58%) of the household heads were in the age group of 31 to 45 years. In addition, 573 (69.45%) household heads were married, 87 (10.06%) had no formal education, and 628 (76.12%) households had four or fewer family members. Furthermore, 160 (19.39%) of the households were classified in the lowest wealth quintile (Table 1).

**Table 1:** Sociodemographic and socioeconomic characteristics of household heads, South Wollo zone, Northeast Ethiopia, 2023.

Variables	Category	Frequency	Percent (%)
Sex of the household head	Male	574	69.58
	Female	251	30.42
Age of the household head	≤30	191	23.15
	31–45	343	41.58
	46–60	182	22.06
	>60	109	13.21
Religion	Orthodox	315	38.18
	Muslim	472	57.21
	Protestant	38	4.61
Marital status of the household head	Single	55	55
	Married	573	69.45
	Separated	28	3.39
	Divorced	74	8.97
	Widowed	95	11.52
Educational status of the household head	No education	83	10.06
	Read and write only	65	7.88
	Primary	108	13.09
	Secondary	134	16.24
	College and above	435	52.73
Occupation of the household head	Unemployed	23	2.79
	Self-employed	376	45.58
	Government employed	385	46.67
	Private employed	41	4.97
Family size	≤4	628	76.12
	>4	197	23.88
Presence of under-five children in the household	Yes	261	31.64
	No	564	68.36
Wealth status	Poorest	160	19.39
	Poorer	131	15.88
	Middle	135	16.36
	Richer	174	21.09
	Richest	225	27.27

### Household annual consumption expenditure

With a standard deviation of 42,488.90, the mean annual household expenditure (food expenditure: ETB 48,160.04 and non-food expenditure: ETB 20,727.27) was ETB 87,827.64. The average annual household health expenditure was ETB 92,41.88, with a standard deviation of 18,923.46 (Table 2).

**Table 2:** Annual expenditure of households in the South Wollo zone, Northeast Ethiopia, 2023.

HH annual expenditure	*N*	Mean (ETB)	Std. Dev
Total household expenditure	825	87827.64	42488.90
Household food expenditure	825	48160.04	20737.03
Non-food household expenditure	825	20727.27	11607.80
Total health expenditure	825	9241.88	18923.46

### Health and health-related characteristics

In 84.24% (695) of the households, one or more members sought modern healthcare. Of these, 5.90% (35) of individuals had a history of referrals. At least one household member utilized traditional healthcare in 21.58% (178) of the households, and 30.18% (249) of the households had at least one member with a chronic health condition (Table 3).

**Table 3:** Health and health-related characteristics of households in the South Wollo zone, Northeast Ethiopia, 2023.

Variables	Category	Frequency	Percent (%)
Modern healthcare seek	Yes	695	84.24
	No	130	15.76
Number of HH members seeking healthcare	Lower	597	85.90
	Higher	98	14.10
Health institution type	Public	460	66.19
	Private	235	33.81
Referral history	Yes	41	5.90
	No	654	94.10
Chronic health conditions	Yes	249	30.18
	No	576	69.82
Seeking traditional healthcare	Yes	178	21.58
	No	647	78.42

### Catastrophic and impoverishing health expenditures

Approximately 30% (247), 11.6% (98), and 11.52% (95) of the households experienced CHE at the 10 and 25% total household expenditure threshold levels and the 40% non-food expenditure threshold level, respectively. Approximately 4% (31) of the households were pushed below the $2.15 (ETB118.25) extreme poverty line after paying for healthcare services. Amounts of ETB 8,345.21 and ETB 10,935.67 were needed to bring the poor households to the poverty line before and after healthcare expenditure, respectively. An additional ETB 2,590.46 was needed to bring the poor households to the poverty line after spending on healthcare services (Table 4).

**Table 4:** Incidence of catastrophic and impoverishing health expenditures, South Wollo zone, Northeast Ethiopia, 2023.

Variables	Measurements	At 10% threshold	At 25% threshold	At 40% threshold
CHE	Catastrophic headcount (%)	29.94	11.6	11.52
	Catastrophic overshoot	19.90	5.32	10.20
	Mean positive gap (%)	66.47	44.71	88.54

	Measurements	Prepayment	Post-payment	Discrepancy
IHE	Poverty headcount (%)	84.36	89.33	3.97
	Poverty gap	8345.21	10935.67	2590.46(31.04%)
	Normalized poverty gap	70.57	92.47	21.90

CHE, Catastrophic Health Expenditure; IHE, Impoverishing health expenditure.

### Coping mechanisms of healthcare expenditure

Of the households, 99% relied on their personal funds to cover their medical expenses. In addition, borrowing and selling household assets were the coping mechanisms used by 4.48 and 2.91% of the households, respectively, for their medical expenditures. It was found that approximately 23.52% of the households were insured under the Community-Based Health Insurance (CBHI) scheme (Table 5).

**Table 5:** Households’ coping mechanisms for healthcare costs, South Wollo zone, Northeast Ethiopia, 2023.

	Category	Frequency	Percent (%)
Insurance status	Insured	194	23.52
	Not insured	631	76.48
Main source of funds for healthcare costs	Own savings	817	99.03
	Social support	140	16.97
	Borrowing	37	4.48
	Selling assets	24	2.91

### Factors associated with catastrophic health expenditure

In the multivariable logistic regression analysis, the age of the household head, marital status of the household head, insurance status of the household, wealth status of the household, presence of chronic health conditions among household members, and seeking traditional healthcare were found to be statistically significant factors associated with CHE (at *p* < 0.05).

The odds of facing CHE among the households headed by individuals older than 60 years were 3.40 times higher (AOR: 3.40, CI: 1.52–7.60) compared to the households headed by individuals aged 30 years or younger. Similarly, the likelihood of CHE was 2.70 times greater (AOR: 2.70, CI: 1.67–4.38) among the uninsured households compared to the insured ones.

The households with at least one member experiencing chronic illnesses were found to be 5.12 times more likely (AOR: 5.12, CI: 3.24–8.10) to face CHE compared to those without such members. In addition, the households with members utilizing traditional healthcare were 2.47 times more vulnerable (AOR: 2.47, CI: 1.60–3.83) to CHE compared to those without members seeking traditional care (Table 6).

**Table 6:** Multivariable regression analysis of catastrophic health expenditure among households, South Wollo zone, Northeast Ethiopia, 2023.

		CHE			
Variables	Category	Yes	No	COR (95%CI)	AOR (95%CI)
Sex of the HH head	Male	187	387	1.54(1.10, 2.16)	1.69(0.88, 3.22)
	Female	60	191	1	1
Age of the HH head	≤30	27	164	1	1
	31–45	93	250	2.26(1.41, 3.62)	1.77(0.97, 3.22)
	46–60	69	113	3.71(2.24, 6.15)	1.91(0.95, 3.86)
	>60	58	51	6.91(3.97, 12.02)	3.40(1.52, 7.60)*
Marital status of the HH head	Single	6	49	1	1
	Married	170	403	3.44(1.44, 8.20)	2.61(0.89, 7.68)
	Separated	5	23	1.76(0.50, 6.42)	2.52(0.59, 10.87)
	Divorced	28	46	4.97(1.88, 13.10)	6.45(1.89, 21.10)*
	Widowed	38	57	5.44(2.12, 13.96)	4.30(1.27, 14.57)*
Family size	≤4	175	453	1	1
	>4	72	125	1.49(1.06, 2.09)	0.97(0.61, 1.56)
Wealth status	Poorest	71	89	4.81(2.95, 7.83)	4.80(2.61, 8.86)*
	Poorer	45	86	3.16(1.88, 5.30)	3.91(2.02, 7.54)*
	Middle	40	95	2.53(1.50, 4.30)	2.42(1.28, 4.59)*
	Richer	59	115	3.10(1.90, 5.04)	2.10(1.16, 3.85)*
	Richest	32	193	1	1
Insurance status	Insured	46	148	1	1
	Non-insured	201	430	1.50(1.04, 2.18)	2.70(1.67, 4.38)*
Chronic health conditions	Yes	155	94	8.67(6.18, 12.18)	5.12(3.24, 8.10)*
	No	92	484	1	1
Seeking traditional medicine	Yes	77	101	2.14(1.52, 3.02)	2.47(1.60, 3.83)*
	No	170	477	1	1
Social support	Yes	77	63	1	1
	No	170	515	0.27(0.17, 0.39)	1.00(0.68, 1.46)
Number of members seeking care	Lower	201	396	1	1
	Higher	46	52	1.74(1.13, 2.68)	1.62(0.94, 2.83)

* Means significant at *p* < 0.05. HH, Household; CHE, Catastrophic Health Expenditure.

Moreover, the poorest households were 4.80 times more likely (AOR: 4.80, CI: 2.61–8.86) to encounter CHE compared to the wealthiest households. Similarly, the households headed by divorced or widowed individuals were found to be more vulnerable to CHE, with odds ratios of 6.45 (AOR: 6.45, CI: 1.89–21.10) and 4.30 (AOR: 4.30, CI: 1.27–14.57), respectively, compared to those headed by single individuals (Table 6).

### Socioeconomic inequality in financial hardship of health expenditure

**Figure 3:**
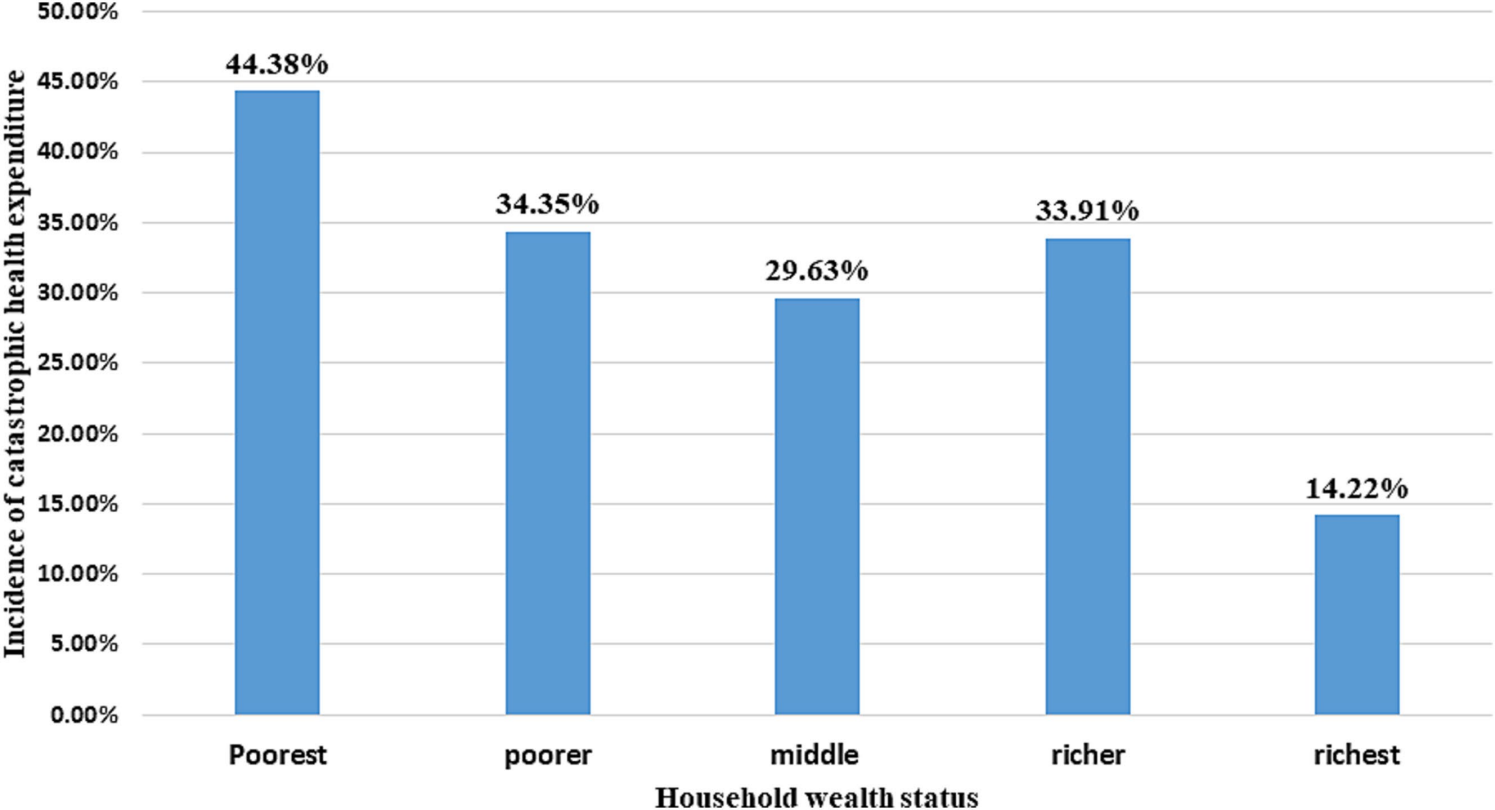
Incidence of catastrophic health expenditure across the wealth categories of households, South Wollo zone, Northeast Ethiopia, 2023.

The magnitude of CHE per household wealth category is shown in Figure 3. Nearly half (44.3%) of the households in the lowest-wealth quintile experienced CHE, compared to less than one-fifth of the households in the richest category (14.22%) who experienced CHE. The graph also shows that the incidence of CHE decreased as the wealth category moved from the poorest to the richest quintile (Figure 3).

There was significant inequality in the incidence of CHE across the socioeconomic groups. The relative CIX value for the incidence of CHE is outlined in Table 7. The estimated CIX for wealth status was −0.17 (95% CI: −0.23, −0.11), with a significant *p*-value (*p* < 0.001), underscoring the significance of the CIX across the different wealth quintiles. A negative CIX value suggested a more prominent concentration of CHE among the poorest households compared to the wealthier ones (Table 7).

**Table 7:** Socioeconomic inequality in financial hardships of health expenditure, South Wollo zone, Northeast Ethiopia, 2023.

Parameter	Observation	CIX	*p*-value	95%CI
CHE	825	−0.17	0.000	(−0.26, −0.11)*
IHE	825	−0.01	0.117	(−0.03, 0.003)

CIX, Concentration index; * significant inequality at *p* < 0.001.

**Figure 4:**
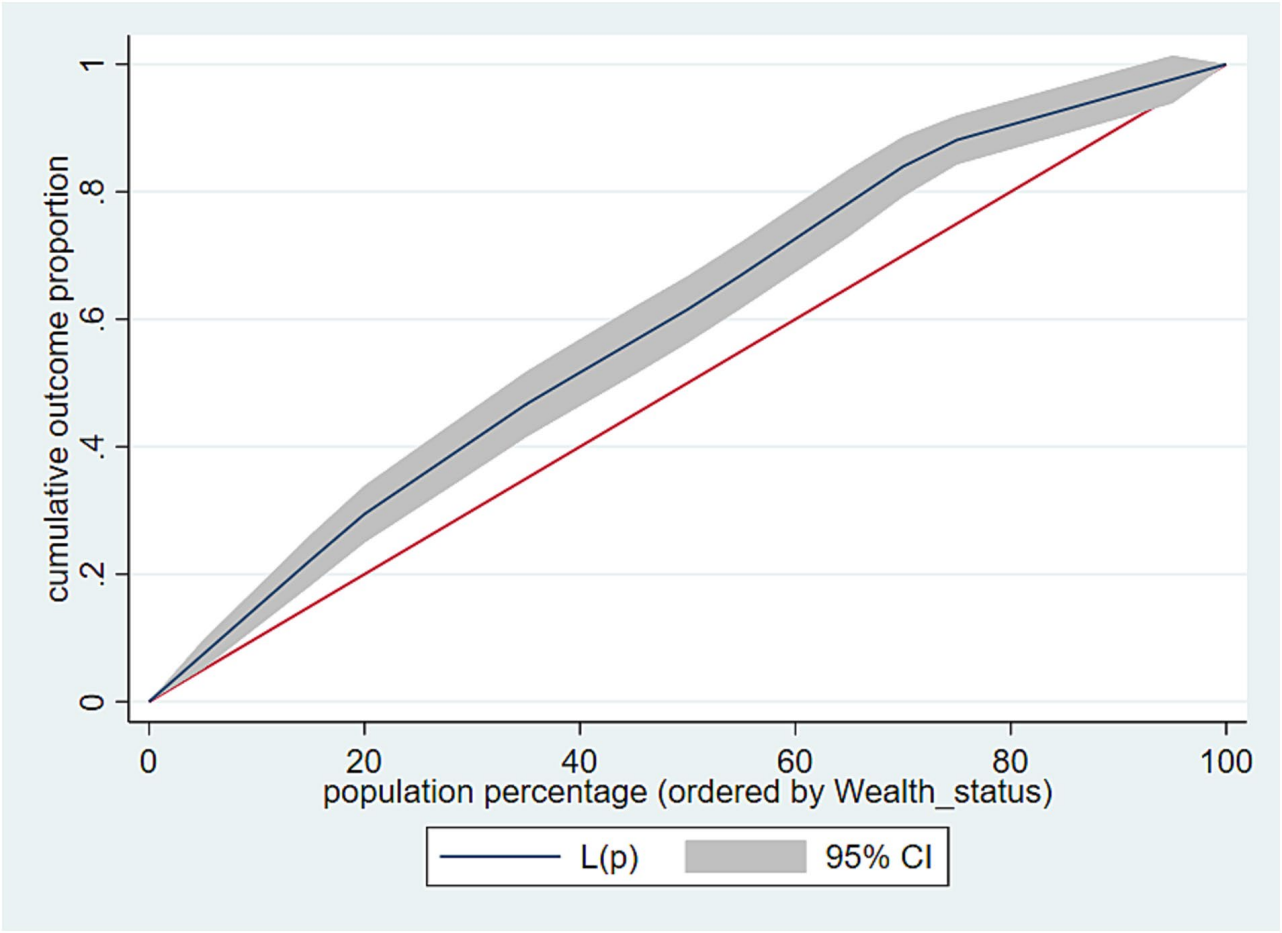
Concentration curve for socioeconomic inequality in Catastrophic Health Expenditure, South Wollo zone, Northeast Ethiopia, 2023.

Furthermore, the concentration curve, positioned above the line of equality, reveals a higher concentration of CHE among the most disadvantaged groups (i.e., the poorest households) (Figure 4).

## Discussion

This study aimed to assess the incidence, determinants, and socioeconomic inequality in financial hardships in accessing quality health services among households in the South Wollo zone. The study revealed that the incidence of CHE and IHE was ~30% and ~ 4%, respectively. This finding implies that a significant number of households are experiencing financial hardships due to paying for healthcare and that preventing IHE could reduce overall impoverishment by nearly 4%. Moreover, the less privileged groups, such as those without insurance, older adults, households with chronically ill members, households headed by divorced or widowed individuals, and users of traditional healthcare services, are particularly vulnerable.

This study also showed that there was significant inequality in the incidence of CHE across the socioeconomic categories, with a high concentration of CHE among the poorest households (31). This finding is supported by the results of studies conducted in Malawi (36) and India (37). This implies that recognizing the high concentration of CHE among the poorest households calls for targeted policy initiatives to protect them from the financial hardships of health expenditure.

The incidence of CHE in the current study was higher compared to the national incidence documented in a previous study, which reported a 2.1% incidence rate (38). This discrepancy may be attributed to variations in the study context; our research comprised indirect medical costs, which were not considered in the previous study. In addition, the discrepancy may be attributed to the use of secondary data in the previous study (2015/16 HCE and WM survey), which could have been influenced by temporal variations.

Furthermore, the incidence of CHE in this study was higher than the incidences reported in prior research on households affected by depression (2019) and severe mental health disorders (SMD) in rural Ethiopia (2015), where the incidences were 20 and 20.3%, respectively, using a 10% threshold level (35, 39). This discrepancy may be attributed to the use of primary data in the current study, which is more recent compared to the studies conducted before 2015.

However, the incidence of CHE in the current study was lower than that reported in a study on the economic burden of diabetes mellitus care among diabetic patients with regular follow-up at public hospitals in Bahir Dar city in 2020, which reported an incidence of CHE at 74.3%, using a 10% threshold level (13). This variation may be explained by the inclusion of insured households and non-ill household members in the current study, potentially reducing the incidence of CHE. This implies that households with a member experiencing chronic conditions are more susceptible to CHE.

Similarly, the incidence of CHE in this study was lower than that reported in previous observational studies, such as a study investigating financial risks associated with maternal and neonatal healthcare in southern Ethiopia in 2020 (CHE incidence: 46% with a 10% threshold level) and a study conducted in Debre Tabor in 2022 at the household level, which indicated an incidence of CHE of 37.1% (11, 40). This variance could potentially originate from the previous study’s use of a prospective cohort design, in contrast to the current study’s application of a cross-sectional approach and the scope of the study. In addition, it might reflect the increased healthcare needs for mothers and newborns compared to other segments of the community (11, 40).

The incidence of CHE in this study was higher than that reported in household-level studies conducted in African countries such as Kenya, Uganda, Morocco, and South Africa, where the incidences of CHE (using a 10% threshold level) were 10.7, 14.2, 12.8, and 9.97%, respectively (41–44). The probable reason for the discrepancy might be due to differences in the study scope, context, sociodemographic and socioeconomic characteristics, and the utilization of secondary data extracted from corresponding nationally representative surveys. The increased cost of healthcare services and political instability in Ethiopia may be other potential factors contributing to the discrepancy.

The incidence of CHE in our study was higher than that reported in the global financial protection monitoring reports from 2019 and 2021, which reported incidences of 12.7 and 13%, respectively (3, 4). This difference might be attributed to variations in the study’s scope and context. Moreover, the global reports heavily relied on secondary data from national reports, which could contribute to the observed variance.

The incidence of IHE in our study (IHE: 3.97%) was higher than the incidence reported in similar studies in Ethiopia, such as a study conducted nationally in 2020, which reported an IHE incidence of 0.9% and research on financial risks associated with seeking maternal and neonatal care in southern Ethiopia, which reported an IHE incidence of 0.3% (38, 40). Similarly, the incidence of IHE in the current study was higher than the findings from studies conducted in Uganda (IHE: 2.7%) and Morocco (IHE: 1.11%) (41, 42). However, the current study’s finding was lower than that of a study conducted on diabetic mellitus patients in Bahir Dar city public hospitals, which reported an IHE value of 5% (13). This discrepancy might be due to the fact that our study included all household members, standardized according to adult equivalent size based on sex and age, unlike the previous studies that focused on specific diseases.

In addition, the households headed by individuals aged 60 years and older exhibited a higher propensity to experience CHE. This finding aligns with the results from studies on CHE among individuals with severe mental disorders in rural Ethiopia and Kenya in 2018 (35, 43). Similarly, the households without insurance coverage had a higher risk of experiencing CHE. This is supported by a study conducted in Kutaber district (45) and highlights the protective role of health insurance in shielding households from healthcare-related financial risks. This finding is further supported by a study conducted in Kenya in 2018, which indicated that households with at least one member enrolled in health insurance were less susceptible to CHE (43).

Moreover, the presence of chronic health conditions within households showed a significant and direct association with CHE. This suggests that chronic health conditions are the main source of financial risk related to healthcare expenses. This conclusion is supported by evidence from studies conducted in Ethiopia, Kenya, and South Africa (35, 39, 41, 46).

In addition, this study revealed that the households headed by widowed and divorced individuals were more likely to experience CHE than the households headed by single individuals. This finding aligns with that of a study conducted in the United States, which stated that widowhood significantly increases the risk of poverty and financial hardship, leading to higher incidences of CHE (47), and a study conducted in Tanzania, which found that widowed and divorced women are more likely to be poor and face economic challenges, making them more susceptible to CHE (48).

Similarly, this study found that the households utilizing traditional healthcare were more vulnerable to CHE. The finding aligns with those of studies conducted in Ethiopia (11, 49) and Malawi (50), which stated that households that utilize traditional healthcare methods are often more vulnerable to CHE. This is partly because traditional treatments can be costly and are typically paid out-of-pocket, which can quickly accumulate and surpass the household’s financial capacity.

### Policy and practical implications

Policymakers should prioritize targeted interventions to protect vulnerable groups, such as older adults, non-insured individuals, and households with chronic illnesses, who face a disproportionately higher risk of financial hardship in healthcare. Expanding social protection programs and universal health insurance coverage, particularly for the poorest households, could significantly reduce the overall burden of impoverishment. In addition, addressing the inequality in CHE across socioeconomic groups is crucial, requiring policies that specifically target the poorest households. Effective chronic disease management programs could also help mitigate the financial risks associated with healthcare.

### Limitations of the study

The main limitation of this study is recall bias. Although measures such as cross-verifying self-reported health expenses with recipients were implemented to mitigate this bias, it remains a concern. Another limitation is the cross-sectional study design, which does not establish a temporal relationship between the outcome variable and explanatory variables.

## Conclusion

This study revealed significant socioeconomic inequality in healthcare-related financial hardship and the number of households in the South Wollo zone experiencing such hardship. The incidence of CHE in this study was higher compared to that reported in previous studies conducted at the household level in Ethiopia. Moreover, the age and marital status of the household head, the insurance status of the household, the presence of chronic health conditions, the use of traditional medicine, and the wealth status of the household were found to be the determinant factors of CHE.

## Data Availability

The original contributions presented in the study are included in the article/supplementary material, further inquiries can be directed to the corresponding author.

## Footnotes

^1^ http://www.statsethiopia.gov.et/population-projection/
